# Systematic Analysis of Alkaline/Neutral Invertase Genes Reveals the Involvement of Smi-miR399 in Regulation of *SmNINV3* and *SmNINV4* in *Salvia miltiorrhiza*

**DOI:** 10.3390/plants8110490

**Published:** 2019-11-11

**Authors:** Hong Zhou, Caili Li, Xiaoxiao Qiu, Shanfa Lu

**Affiliations:** Institute of Medicinal Plant Development, Chinese Academy of Medical Sciences & Peking Union Medical College, No.151 Malianwa North Road, Haidian District, Beijing 100193, China; zhouhong1013@126.com (H.Z.); clli@implad.ac.cn (C.L.); 18813756675@163.com (X.Q.)

**Keywords:** Smi-miR399, alkaline/neutral invertase, *PHO2*, *Salvia milltiorrhiza*

## Abstract

Alkaline/neutral invertases (*NINVs*), which irreversibly catalyze the hydrolysis of sucrose into fructose and glucose, play crucial roles in carbohydrate metabolism and plant development. Comprehensive insights into *NINV* genes are lacking in *Salvia miltiorrhiza*, a well-known traditional Chinese medicinal (TCM) plant with significant medicinal and economic value. Through genome-wide prediction, nine putative *SmNINV* genes, termed *SmNINV1*-*SmNINV9*, were identified. Integrated analysis of gene structures, sequence features, conserved domains, conserved motifs and phylogenetic trees revealed the conservation and divergence of *SmNINVs*. The identified *SmNINVs* were differentially expressed in roots, stems, leaves, flowers, and different root tissues. They also responded to drought, salicylic acid, yeast extract, and methyl jasmonate treatments. More importantly, computational prediction and experimental validation showed that *SmNINV3* and *SmNINV4* were targets of Smi-miR399, a conserved miRNA previously shown to affect Pi uptake and translocation through the cleavage of *PHOSPHATE2* (*PHO2*). Consistently, analysis of 43 *NINV* genes and 26 miR399 sequences from *Arabidopsis thaliana*, *Populus trichocarpa*, *Manihot esculenta,* and *Solanum lycopersicum* showed that various *AtNINV*, *PtNINV*, *MeNINV,* and *SlNINV* genes were regulated by miR399. It indicates that the miR399-*NINV* module exists widely in plants. Furthermore, Smi-miR399 also cleaved *SmPHO2* transcripts in *S. miltiorrhiza*, suggesting the complexity of *NINVs*, *PHO2,* and miR399 networks.

## 1. Introduction

In plants, invertases are pivotal enzymes involved in sucrose metabolism. They catalyze irreversible hydrolysis of sucrose into glucose and fructose [1,2]. Based on the optimum pH, invertases can be divided into two classes, including the acid invertase (AINV) class and the alkaline/neutral invertase (NINV) class. AINVs are β-fructofuranosidases and N-glycosylated proteins with an optimal pH of 4.5–5.5, whereas NINVs consist of a novel family of glucosidases with optimal pH of 6.5–8.0. They are substantially distinct in evolutionary origins, and biochemical and molecular characteristics [3,4]. AINVs evolved from respiratory eukaryotes and aerobic bacteria [5]. They locate in either the cell wall or the vacuoles [6,7], indicating the role of AINVs in source-sink interactions and in plant responses to environmental cues via sugar metabolism and signaling pathways [8,9,10,11,12]. In contrast to AINVs, NINVs originated from cyanobacteria and are unique to plants and photosynthetic bacteria [13]. They locate in the cytosol and in multiple subcellular compartments, including the mitochondria, the chloroplasts, and the nucleus [14,15,16]. Since NINVs are not glycosylated [17], they are less stable and have lower enzymatic activity compared with AINVs. It results in lack of knowledge on their functions over the past decades [3,13].

With the advent of whole-genomic sequencing projects, genes coding for NINV proteins have recently been identified in several species, such as *Arabidopsis thaliana* [18], *Populus trichocarpa* [19], *Oryza sativa* [20], *Glycine max* [21], *Manihot esculenta* [22], *Camellia sinensis* [23], and *Solanum lycopersicum* [24]. Functional analysis showed that NINVs played significant roles in plant growth, particularly in the development of roots and reproductive organs. Mutation of *Arabidopsis AT1G35580* (*A/N-InvG*) caused earlier floral transition, short primary roots, and smaller rosette leaves and siliques [16,25]. Double mutation of *AT1G35580* and *AT4G09510* (*A/N-InvI*) resulted in drastically reduced root growth and abnormal cell enlargement [26]. Point-mutation of *AT5G22510* (*A/N-InvE*) inhibited photosynthetic apparatus development and enhanced nitrate assimilation in *Arabidopsis* seedlings treated with sugar [27,28]. Mutation of *AT3G05820* (*A/N-InvH*) caused severe reduction of shoot growth and delay in the first stage of flowering [29]. Similarly, the loss-of-function mutation of *Lotus japonicus LjINV1* [30] and rice *OsCyt-INV1* [31] reduced root growth and impaired pollen development and flowering. In addition to plant development, NINVs are also involved in plant responses to environmental stresses, such as osmotic stress and cold stress, through controlling sugar accumulation and respiration, altering osmotic potential, and activating sugar signaling pathways [25,32]. Despite of their important role, our current knowledge on the *NINV* gene family is limited to a small number of plant species. Functional characterization of *NINVs* has mainly concentrated on *Arabidopsis*. *NINV* genes in *S. miltiorrhiza* have not been identified. In addition, there is no information on miRNA-mediated posttranscriptional regulation of *NINV* genes.

MiR399 is a well-documented and deeply conserved miRNA. It is known to be involved in plant responses to inorganic phosphate (Pi) starvation by cleavage of *PHO2* transcripts in plants [33,34,35,36]. Under low Pi conditions, up-regulation of miR399 was accompanied by down-regulation of *PHO2* and improvement of Pi uptake and translocation. In addition to Pi stress, recent studies showed that miR399 was also involved in plant responses to the starvation of multiple nutrients, such as iron (Fe), potassium (K), sodium (Na), and calcium (Ca) [37]. In the other studies, the miR399-*PHO2* module was found to affect flowering time in response to ambient temperature changes [38]. These results suggest functional diversity and complexity of miR399 in plants. To date, the regulatory role of miR399 in NINVs has not been demonstrated.

*Salvia miltiorrhiza* Bunge belongs to the genus *Salvia* of the family Lamiacae. Its dried roots and rhizomes are well-known materials of traditional Chinese medicines widely used in treating cardiovascular and cerebrovascular diseases [39]. *S. miltiorrhiza* is also an emerging model system in medicinal plant biology. It suggests that this plant species has high economic and scientific value [40]. However, the yield and quality of *S. miltiorrhiza* are often affected by environmental stresses, such as drought, salinity, and temperature [41]. Based on current knowledge of *NINVs*, we presume that *NINVs* play significant roles in the growth, development and stress responses of *S. miltiorrhiza*. However, comprehensive insights into *S. miltiorrhiza SmNINV* genes are lacking. Thus, we carried out genome-wide identification and characterization of *SmNINV* genes. Comparative analysis of *SmNINVs* and *NINVs* from other plant species showed conservation and divergence of *NINVs*. *SmNINVs* were differentially expressed in various *S. miltiorrhiza* organs and root tissues and responded to drought, salicylic acid (SA), yeast extract (YE), and methyl jasmonate (MeJA) treatments. Interestingly, computational analysis of sRNAome and degradome and 5′ RACE experimental validation showed that, except for *PHO2*, the expression of *SmNINV3* and *SmNINV4* were also posttranscriptionally regulated by Smi-miR399. The results provide the first hand of information for further elucidating the potential functions and post-transcriptional regulatory mechanism of *NINV* genes in *S. miltiorrhiza.*

## 2. Results

### 2.1. Genome-Wide Identification and Characterization of the SmNINV Gene Family

**Figure 1:**
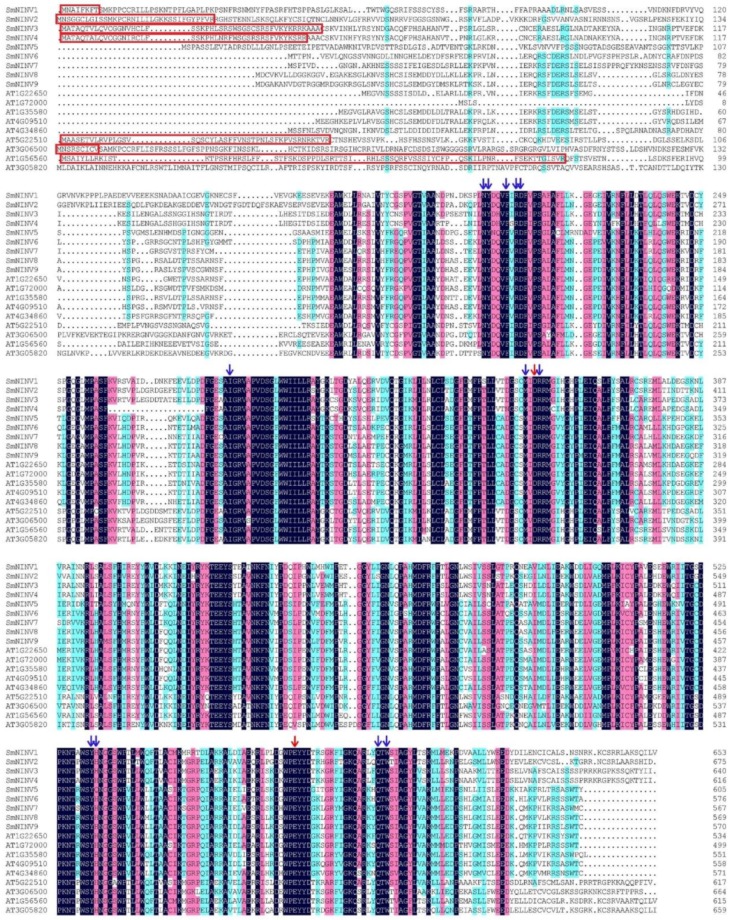
Amino acid sequence alignment of eighteen neutral/alkaline invertases in *S. miltiorrhiza* and *Arabidopsis*. Dark-blue, violet and light blue shadings reflect 100%, 75%, and 50% amino acid conservation, respectively. Catalytic residues and substrate-binding residues are indicated by red and blue arrows, respectively. Signal peptide sequences predicted by Target P 1.1 were marked by red boxes.

In order to identify *SmNINV* genes, BLAST analysis of *Arabidopsis* AtNINVs proteins against the current assembly of the *S. miltiorrhiza* (line 99–3) genome was carried out [42,43]. After removing redundant sequences, nine putative *NINV* genes were identified. They were named *SmNINV1* to *SmNINV9*, respectively. The number of identified *SmNINV* genes is comparable with that in other plant species, such as *A. thaliana* [18], *C. sinensis* [23], and *S. lycopersicum* [24]. *A. thaliana* has 9 *NINVs*, *C. sinensis* contains 8, and *S. lycopersicum* has 7. The length of *SmNINV* open reading frames (ORFs) varies from 1704 to 2028 bp. The deduced proteins have 567 to 675 amino acids. The theoretical isoelectric point varied from 5.53 to 8.67. The molecular weight varied from 64.61 to 76.15 kDa. The detailed features of *SmNINVs* were listed in Table 1. Sequence alignment showed that SmNINV and AtNINV proteins had relatively higher similarity at the C-terminus than the N-terminus (Figure 1). Similar to pepper *CaNINVs* [44], *SmNINVs* have multiple conserved residues. It includes substrate-binding residues D351 and E577 and catalytic residues N204, Y205, F209, R211, D212, I285, M349, R352, Y533, H534, Q595, and W597 (the numbers were based on *SmNINV1*) (Figure 1). Computational prediction of subcellular localization signals was performed by Target P 1.1. The results showed that *SmNINV1*–*SmNINV4* is possibly located in the chloroplasts (the predicted signal peptide sequences were marked by red boxes in Figure 1), whereas the other *SmNINVs* are likely located in the places other than the chloroplasts, the mitochondria and the secretory pathways (Table 1).

### 2.2. Structure and Phylogenetic Analysis of SmNINVs

**Figure 2:**
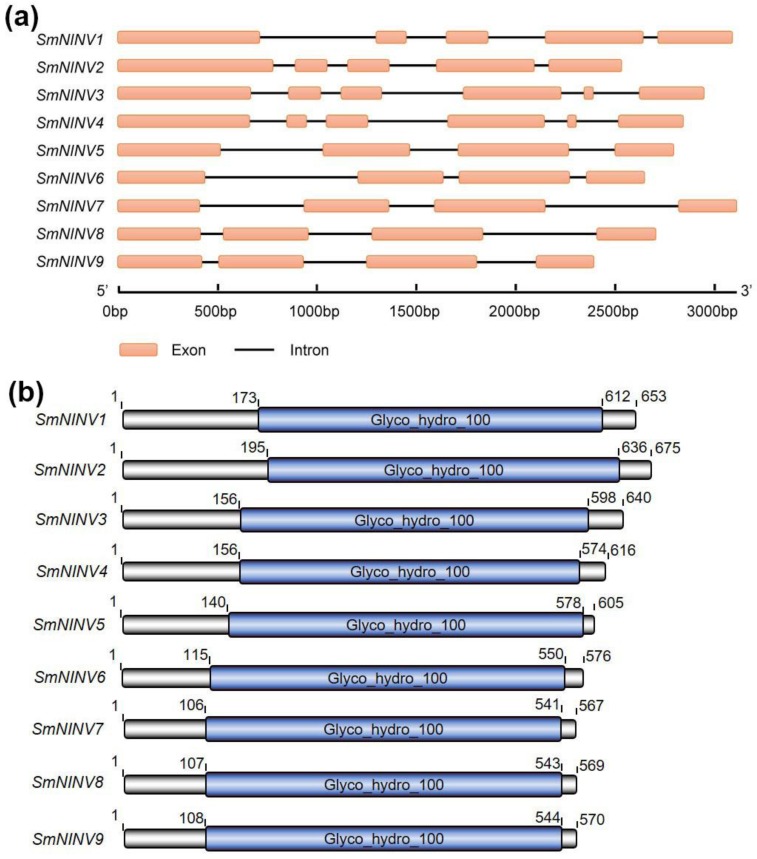
Sequence feature of NINVs in *S. miltiorrhiza*. (**a**) Exon–intron structure of nine *SmNINVs*. Exons and introns are shown. (**b**) Conserved domains in SmNINVs. Conserved domains were predicated by searching Pfam and shown in blue boxes. Names of conserved domains are indicated.

In this study, we identified nine *SmNINV* genes. Sequence alignment of *SmNINV* cDNAs and genomic sequences [43] showed that the number of exons of *SmNINV* genes varied from four to six. Among the nine identified genes, *SmNINV1* and *SmNINV2* have five exons. *SmNINV3* and *SmNINV4* share similar structures. Both of them have six exons, of which the fifth is the smallest. The number of introns for *SmNINV5*–*SmNINV9* is four (Figure 2a). The deduced amino acid sequences show high similarities to NINV proteins from other plant species. All of them contain the conserved domain, Glyco_hydro_100 (PF12899.7) (Figure 2b).

**Figure 3:**
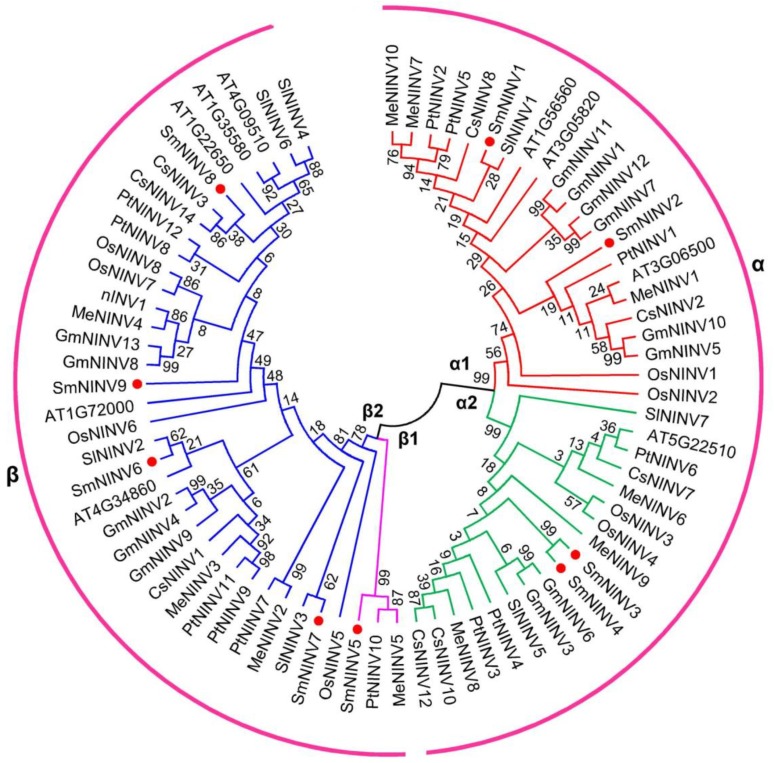
Phylogenetic analysis of 77 alkaline/neutral invertase (NINV) proteins from eight plant species. The phylogenetic tree was constructed by the neighbor-Joining method (1000 bootstrap replicates) using MEGA version 7.0 [45]. Red dots indicate NINVs from *S. miltiorrhiza.* Groups and subgroups are indicated by α/β and α1/α2/β1/β2, respectively.

In order to determine the evolutionary relationships among SmNINVs, we constructed a neighbor-joining (NJ) phylogenetic tree for 77 full-length proteins from eight plant species. Based on the NJ tree, NINVs can be classified into two main groups, including the α group and the β group (Figure 3). It is consistent with previous studies [13]. Four SmNINVs, including SmNINV1–SmNINV4, which carry a chloroplast transit peptide (Table 1), belong to α group (Figure 3). The other five SmNINVs, including SmNINV5–SmNINV9, which lack any subcellular targeting signals (Table 1), belong to β group (Figure 3). In the α group, SmNINV1 and SmNINV2, which share 78.76% amino acid identity, belong to α1 subgroup. SmNINV3 and SmNINV4, which share 94.97% amino acid identity, are members of α2 subgroup. In the β group, SmNINV5 has a close relationship with PtNINV10 and MeNINV5. They are clustered into β1 subgroup. SmNINV6 shares 67% and 88.74% amino acid identity with SlNINV2 and AT4G34860 (A/N-InvB), respectively, and SmNINV7 shares 81.66% amino acid identity with SlNINV3. These genes, together with SmNINV8 and SmNINV9, are included in β2 subgroup.

**Figure 4:**
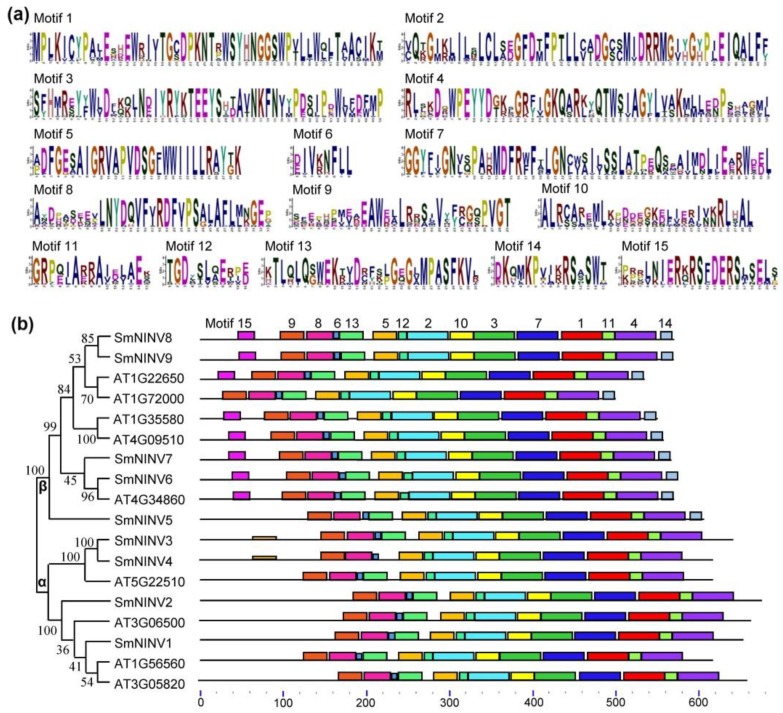
Distribution of conserved motifs of NINV proteins from *S. miltiorrhiza* and *A. thaliana*. (**a**) Sequence logos of 15 conserved motifs in NINVs. The sequences were identified and the logos were created using MEME. (**b**) Architecture of conserved motifs in SmNINVs and AtNINVs. The neighbor-joining (NJ) tree was constructed with full-length amino acid sequences of SmNINVs and AtNINVs using MEGA7 software [45] with 1000 bootstraps. Each motif is indicated by a colored box. Box size indicates the length of motifs. Phylogenetic groups and the number of motifs are shown.

To further determine the relationships of plant NINVs, we analyzed putative motifs of SmNINVs and AtNINVs. A total of fifteen distinct motifs were identified (Figure 4a). SmNINVs and AtNINVs share similar distribution patterns of motifs (Figure 4b). It indicates that SmNINVs and AtNINVs have close evolutionary relationships. Most of the motifs are highly conserved and widely distributed in all of the analyzed NINV proteins. However, there are several exceptional cases (Figure 4b). For instance, motifs 14 and 15 are specifically distributed in β group NINVs, although motif 15 does not exist in AT1G72000 (A/N-InvF) and SmNINV5. In addition, SmNINV3 and SmNINV4, which belong to α group, contain two motif 5s. Motif 13 is absent in SmNINV4. Additionally, except for motif 14 and 15, other 13 motifs are overlaid with the conserved domain Glyco_hydro_100 (PF12899.7). These results suggest the conservation and divergence of plant NINVs.

### 2.3. Expression Patterns of SmNINVs in Different Organs and Root Tissues

**Figure 5:**
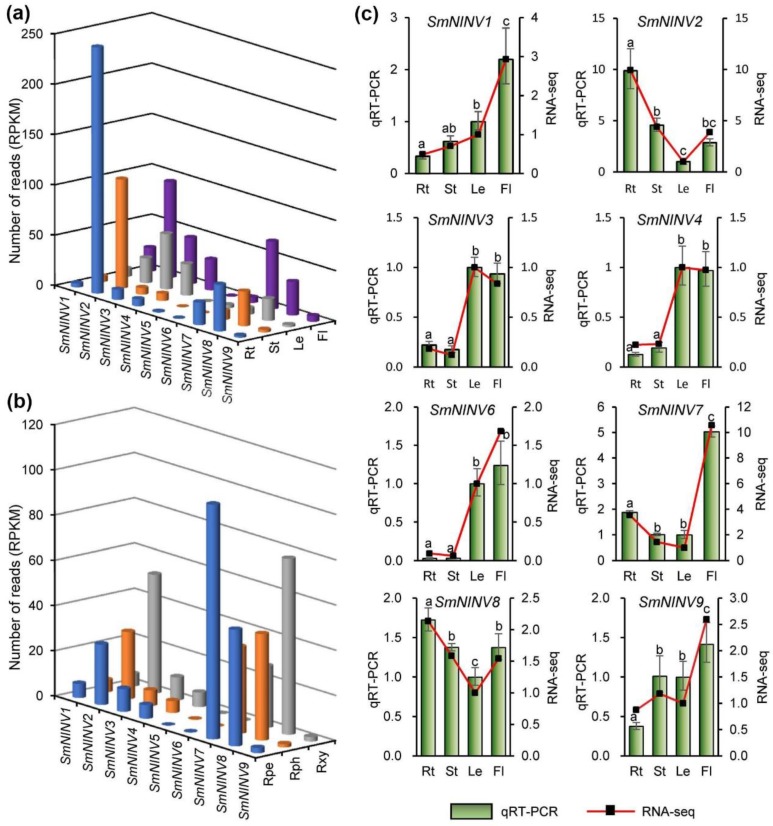
Expression of *SmNINVs* in *S. miltiorrhiza*. (**a**) Number of reads per kilobase per million mapped reads (RPKM) of *SmNINVs* in RNA-seq data from roots (Rt), stems (St), leaves (Le), and flowers (Fl). (**b**) Number of reads (RPKM) of *SmNINVs* in RNA-seq data from root periderm (Rpe), root phloem (Rph) and root xylem (Rxy). (**c**) Relative expression of *SmNINVs* in roots (Rt), stems (St), leaves (Le) and flowers (Fl) of *S. miltiorrhiza*. Expression level in leaves was arbitrarily set to 1 and the levels in other organs were given relative to this. One-way ANOVA was calculated for qRT-PCR data using IBM SPSS 20 software. *P* < 0.05 was considered statistically significant and was represented by different letters. The bars represent standard errors. Fold changes of *SmNINV* expression in RNA-seq are shown by the red lines.

*NINV* genes are important for plant growth and development [16,25,26,29,30,31]. In order to preliminarily elucidate the function of *SmNINVs* in *S. miltiorrhiza*, we analyzed their expression patterns using RNA-seq data from stems, leaves, flowers, whole roots, root periderm, root phloem, and root xylem (Figure 5a,b, Tables S1 and S2). We also examined the expression of nine *SmNINVs* in roots, stems, leaves and flowers of *S. miltiorrhiza* (line 99–3) plants using qRT-PCR (Figure 5c). Overall, *SmNINVs* were differentially expressed, and the results from RNA-seq data and qRT-PCR showed similar trends for each *SmNINV* (Figure 5a,c). *SmNINV1* was mainly expressed in flowers. Its expression in whole roots was relatively low. *SmNINV2* showed relatively high expression in most of the analyzed organs and root tissues with the highest in whole roots. *SmNINV3*, *SmNINV4* and *SmNINV6* showed similar expression patterns with relatively high levels in leaves and flowers. However, the overall expression level of *SmNINV6* is low compared with *SmNINV3* and *SmNINV4*. Very low expression levels were also found for *SmNINV5* and *SmNINV9* in all of the organs and root tissues analyzed. No RNA-seq reads were identified for *SmNINV5* in root periderm, and the expression of *SmNINV5* could not be detected using qRT-PCR. *SmNINV7* and *SmNINV8* were highly expressed in flowers and whole roots. Among different root tissues, *SmNINV7* was predominantly expressed in root periderm, whereas *SmNINV8* showed relatively high expression in root xylem. Differential expression of *SmNINVs* indicates that they may be involved in different physiological processes.

### 2.4. Expression of SmNINVs in Response to Drought, Salicylic Acid (SA), Yeast Extract (YE) and Methyl Jasmonate (MeJA) Treatments

**Figure 6:**
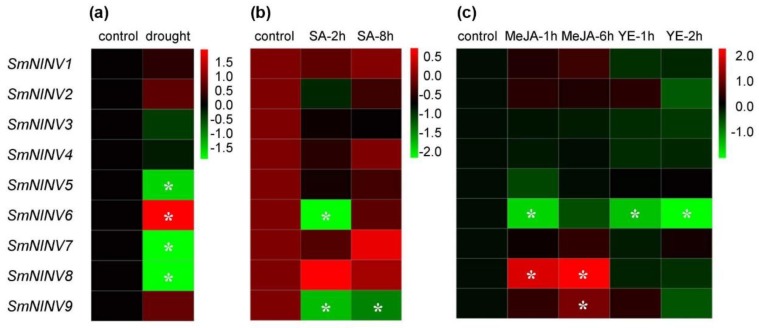
Expression of *SmNINV* genes in *S. miltiorrhiza* under various stresses. (**a**) Responses of *SmNINVs* to drought stress. (**b**) Responses of *SmNINVs* in *S. miltiorrhiza* cell cultures treated with salicylic acid (SA) for 0 (control), 2 (SA-2h), and 8 h (SA-8h). (**c**) Responses of *SmNINVs* in *S. miltiorrhiza* hair roots treated with methyl jasmonate (MeJA) for 0 (control), 1 (MeJA-1h) and 6 h (MeJA-6h) and yeast extract (YE) for 1 (YE-1h) and 2 h (YE-2h). *P* < 0.05 was considered statistically significant. * represents significant differential transcript abundance compared with control.

It has been shown that *NINV* genes are involved in plant responses to environmental stresses [21,23,25,29]. In order to investigate the response of *SmNINVs* under stress conditions, the levels of transcripts in tissues treated with drought, SA, YE and MeJA were analyzed (Figure 6, Tables S3–S5). The results showed that *SmNINV5*, *SmNINV6*, *SmNINV7,* and *SmNINV8* were drought-responsive (Figure 6a, Table S3), *SmNINV6* and *SmNINV9* were SA-responsive (Figure 6b, Table S4), *SmNINV6* was YE-responsive, and *SmNINV6*, *SmNINV8,* and *SmNINV9* were MeJA-responsive (Figure 6c, Table S5). Interestingly, all of these stress-responsive *SmNINVs* belong to β group in the NJ phylogenetic tree (Figure 3). It suggests that the expression of β group *SmNINVs* is more sensitive to the treatments analyzed than α group *SmNINVs*. Among these β group *SmNINVs*, differential responses were observed (Figure 6, Tables S3–S5). For instance, *SmNINV6* was up-regulated under drought stress, whereas it was down-regulated under SA, YE, and MeJA treatments. *SmNINV8* was down-regulated under drought stress. It was up-regulated when treated with MeJA. The results indicate that *SmNINVs* may play different physiological roles under different stress conditions.

### 2.5. MiR399-Mediated Posttranscriptional Regulation of NINVs

**Figure 7:**
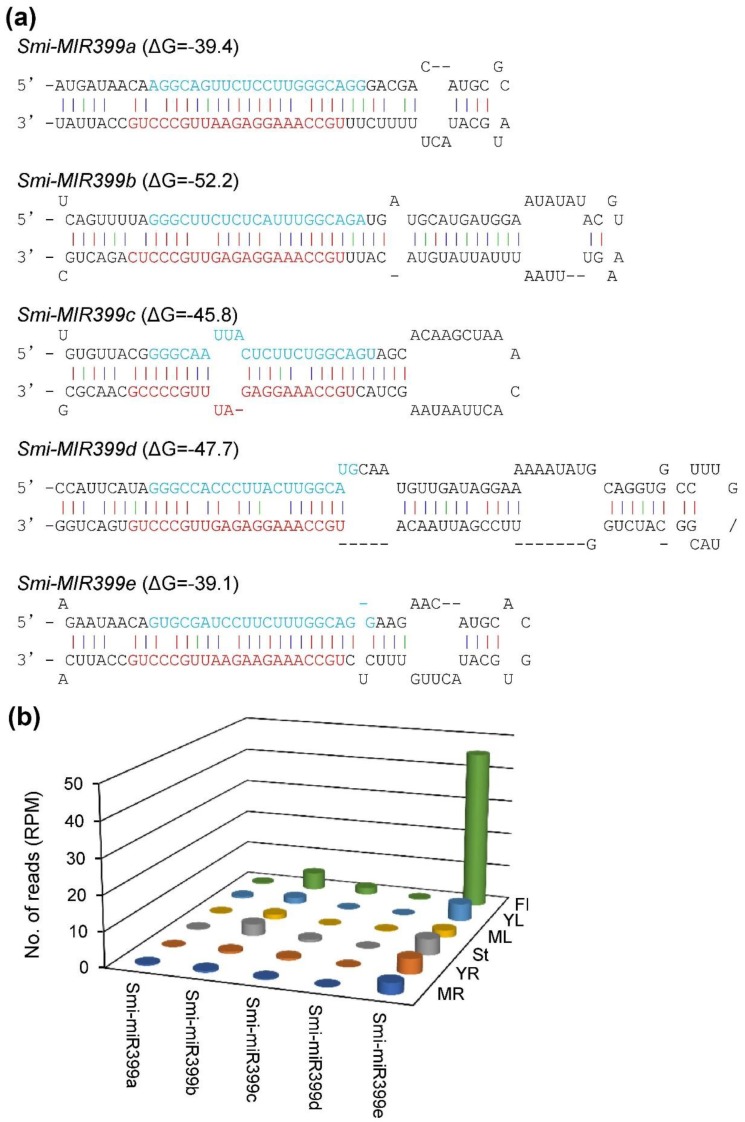
Smi-miR399 in *S. miltiorrhiza*. (**a**) Hairpin structures of *Smi-MIR399* precursors. Mature miRNA sequences are indicated in red. miRNA* sequences are indicated in blue. (**b**) Number of reads per million mapped reads (RPM) of Smi-miR399s in the libraries of small RNAs from mature roots (MR), young roots (YR), stems (St), mature leaves (ML), young leaves (YL), and flowers (Fl).

Plant miRNAs are a class of endogenous small non-coding RNAs with length about 21 nucleotides. They play pivotal roles in a variety of biological processes through regulating gene expression at the posttranscriptional level [46]. In order to elucidate whether *SmNINVs* are regulated by microRNAs, we performed a target search of the *S. miltiorrhiza* sRNA database against nine *SmNINV* cDNA sequences using the online psRNATarget program with maximum expectations of 3.0 [47]. A total of 866 small RNAs were found to have perfect or near-perfect complementarity to *SmNINVs*. The retrieved sRNAs were aligned with the *S. miltiorrhiza* genome (line 99-3). The genome sequence surrounding the small RNAs were predicted for secondary structures using the psRobot software package [48] with the default parameters and checked manually according to the criteria suggested by Meyers et al [49]. Finally, a total of five hairpin structures were identified. Annotation of the resulting miRNAs through BLAST analysis against the miRBase 22.0 [50] showed that these miRNAs were members of the *MIR399* family. They were named Smi-miR399a, Smi-miR399b, Smi-miR399c, Smi-miR399d, and Smi-miR399e, respectively (Figure 7a). Analysis of the high-throughput *S. miltiorrhiza* sRNA database showed that Smi-miR399a and Smi-miR399d were expressed very low in the organs analyzed. Smi-miR399b was predominantly expressed in stems and flowers. Smi-miR399c was predominantly expressed in flowers. Smi-miR399e showed higher levels than other Smi-miR399 members in all of the organs analyzed, with the highest accumulation in flowers (Figure 7b).

**Figure 8:**
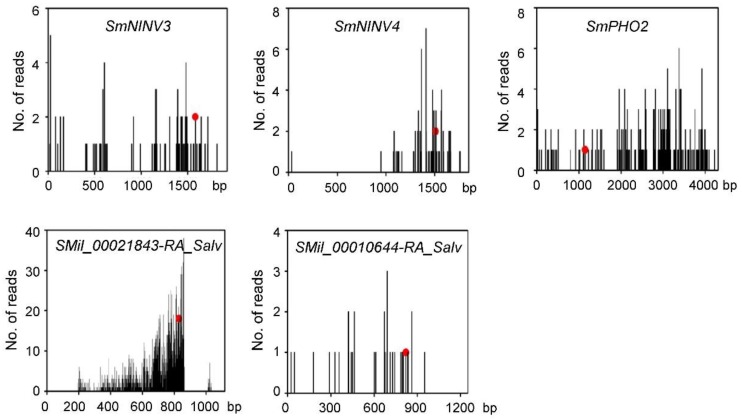
Degradome analysis of Smi-miR399-directed cleavage. X-axis shows the nucleotide (nt) position of gene and Y-axis shows the number of reads obtained by degradome sequencing. Each black line represents a degradome fragment mapped to the genes. The red spots indicate that the products are resulted from Smi-miR399-directed cleavage.

**Figure 9:**
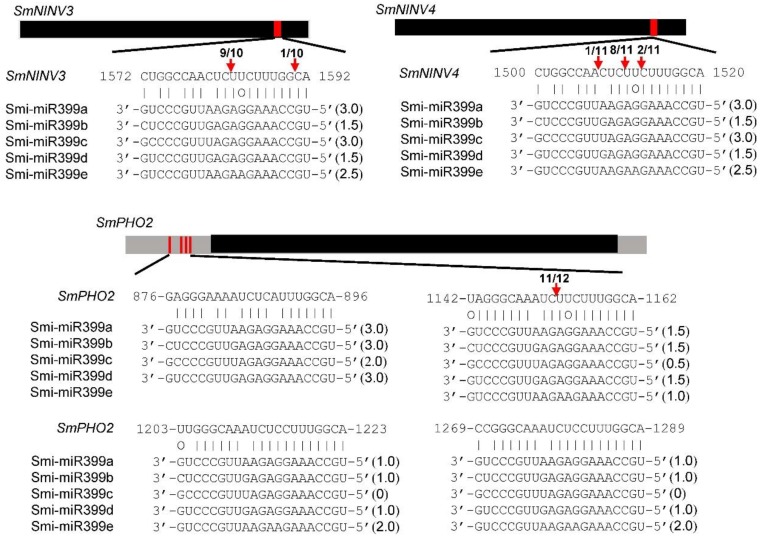
Experimental validation of Smi-miR399-directed cleavage using the 5′ RLM-RACE method. Black lines represent ORFs. Grey lines represent the 5’ and 3’ UTRs. The nucleotide positions of the Smi-miR399 complementary sites (red) of genes are indicated. The mRNA sequence of each complementary site from 5′ to 3′ and the mautre Smi-miR399 sequences from 3′ to 5′ are shown. The expectations predicted using psRNATarget are shown in parentheses. Watson–Crick pairings are indicated by vertical dashes. G:U wobble pairings are indicated by circles. Vertical red arrows indicate the 5′ termini of miR399-directed cleavage products, as identified by 5′ RLM-RACE, with the frequency of clones shown.

It has been shown that miR399 is involved in the regulation of Pi homeostasis through targeting *PHO2* transcripts for cleavage [33,34,35,36]. However, miR399-mediated regulation of *NINV* gene expression was not clear [51], although the *NINV* gene family had been identified in several plant species [18,19,20,21,22,23,24]. Computational target prediction showed that *SmNINV3* and *SmNINV4* contained a region with near-perfect complementarity to Smi-miR399. This region encodes the amino acid sequence WPTLLW, which is absent from other SmNINVs. In order to validate computational prediction, we analyzed degradome data from *S. miltiorrhiza* plants and carried out the modified 5′ RLM-RACE experiments as previously described [52]. The results showed that *SmNINV3* and *SmNINV4* were indeed cleaved by Smi-miR399 (Figure 8 and Figure 9).

**Figure 10:**
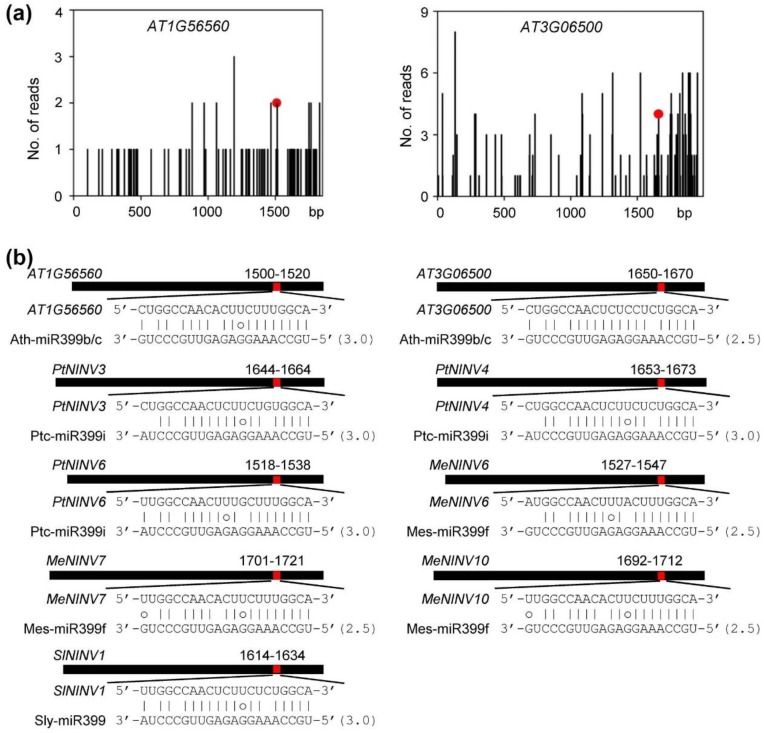
The miR399-*NINV* regulatory module in other plant species. (**a**) Degradome analysis of Ath-miR399b/c-directed cleavage of *AT1G56560* and *AT3G06500*. X-axis shows the nucleotide (nt) position of *NINV* ORFs and Y-axis shows the number of reads obtained by degradome sequencing. Each black line represents a degradome fragment mapped to the ORFs. The red spots indicate that the products are resulted from Ath-miR399b/c-directed cleavage. (**b**) Computational prediction of miR399-directed cleavage of *NINVs* in *A. thaliana* (*At*), *P. trichocarpa* (*Pt*), *M. esculenta* (*Me*), and *S. lycopersicum* (*Sl*). Heavy black lines represent ORFs. The miR399 complementary sites (red) with the nucleotide positions of *SmNINVs* are indicated. The mRNA sequence of each complementary site from 5′ to 3′ and the mautre miR399 sequences from 3′ to 5′ are shown in the expanded regions. The expectations predicted using psRNATarget are shown in parentheses. Watson–Crick pairings are indicated by vertical dashes. G:U wobble pairings are indicated by circles.

The finding of miR399-*NINV* module in *S. miltiorrhiza* raises a question of whether miR399-directed cleavage on *NINV* transcripts is species-specific. In order to address this question, we analyzed the miR399-*NINV* module in other plant species with *NINV* genes and miR399 sequences available, such as *A. thaliana*, *P. trichocarpa*, *M. esculenta*, and *S. lycopersicum*. Computational prediction and degradome (GSM280226 and GSM280227 [36]) analysis showed that *Arabidopsis AT1G56560* (*A/N-InvA*) and *AT3G06500* (*A/N-InvC*) could be targeted by Ath-miR399b/c (Figure 10a,b). In addition, computational prediction showed that *P. trichocarpa PtNINV3*, *PtNINV4* and *PtNINV6* could be regulated by Ptc-miR399i, *S. lycopersicum SlNINV1* could be targeted by Sly-miR399, and *M. esculenta MeNINV6*, *MeNINV7* and *MeNINV10* could be targeted by Mes-miR399f (Figure 10b). It indicates that the miR399-*NINV* module exists not only in *S. miltiorrhiza,* but also in other plant species.

### 2.6. MiR399-Mediated Posttranscriptional Regulation of SmPHO2

MiR399 was reported to regulate *PHO2* genes in various plants [33,34,35,36]. In order to comprehensively understand the role of Smi-miR399 in *S. miltiorrhiza*, we performed a target search of Smi-miR399 against the gene set predicted from *S. miltiorrhiza* genome assembly using psRNATarget with maximum expectations of 3.0 [43,47]. A total of 42 predicted genes were found to have perfect or near-perfect complementarity to Smi-miR399. Degradome data analysis showed that 5 of the 43 genes had degraded cDNA fragments with 5’-end mapped to the predicted Smi-miR399 cleavage sites. It includes *SmNINV3*, *SmNINV4*, *SmPHO2*, an uncharacterized protein gene (SMil_00010644-RA_Salv), and a vacuolar sorting protein gene (SMil_00021843-RA_Salv) (Figure 8). The other 37, which were not further analyzed, could not be verified by the degradome data.

Next, the modified 5′ RLM-RACE method was performed to experimentally validate the cleavage of Smi-miR399 on *SmPHO2*, SMil_00010644-RA_Salv, and SMil_00021843-RA_Salv as previously described [52]. No PCR fragments with the expected size could be obtained for SMil_00010644-RA_Salv and SMil_00021843-RA_Salv. It suggested that the cleavage of Smi-miR399 on SMil_00010644-RA_Salv and SMil_00021843-RA_Salv was not experimentally validated. *SmPHO2* contains four Smi-miR399 complementary sites in the 5′-UTR, one of which was validated to be cleaved by Smi-miR399 (Figure 9). Multiple miR399 complementary sites also exist in other plant *PHO2* [34]. It suggests the conservation of miR399-*PHO2* module in *S. miltiorrhiza* and other plants.

### 2.7. The Effect of Exogenous Sucrose on miR399s, SmNINV3, and SmNINV4 Expression in S. miltiorrhiza

**Figure 11:**
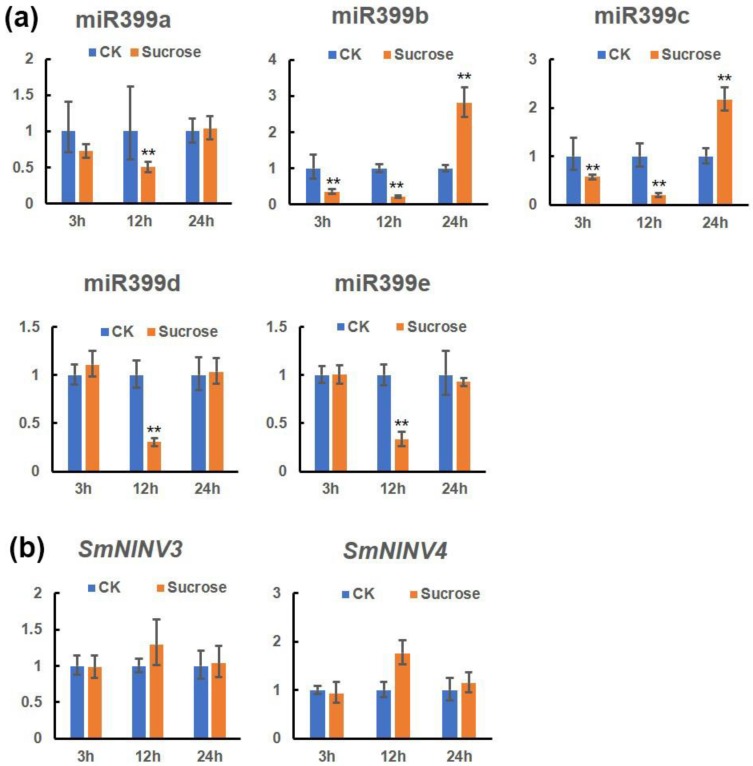
Relative expression levels of miR399a-miR399e, *SmNINV3,* and *SmNINV4* in leaves of *S. miltiorrhiza* subjected to exogenous sucrose treatments. Fold changes of miR399s (**a**), *SmNINV3* and *SmNINV4* (**b**) in leaves of *S. miltiorrhiza* plantlets treated with 3% sucrose for 3, 12, and 24 h are shown. The level of transcripts in leaves treated with Hoagland’s medium (CK) was arbitrarily set to 1 and the levels in leaves treated with Hoagland’s medium containing 3% sucrose were given relative to this. Mean values and standard deviations were obtained from three biological and three technical replicates. ANOVA (analysis of variance) was calculated using SPSS. *P* < 0.05 (*) and *P* < 0.01 (**) were considered statistically significant and extremely significant, respectively.

Both miR399 and *NINVs* were found to be sucrose-responsive. External sucrose supplementation inhibits the expression of miR399 in maize [53]. In addition, the *NINV* expression levels in *Poncirus trifoliata* were induced by sucrose, and decreaed by glucose [54]. To explore the relationships among sucrose, miR399 and *NINVs* in *S. miltiorrhiza*, we determined the expression profiles of Smi-miR399 and its targets, *SmNINV3* and *SmNINV4*, in response to exogenous sucrose supplementation. As shown in Figure 11, the expression levels of all five Smi-miR399s, including Smi-miR399a–Smi-miR399e, were significantly downregulated at 12 h of sucrose treatment. In contrast, the target genes of Smi-miR399, including *SmNINV3* and *SmNINV4*, displayed opposite expression patterns with the levels induced at 12 h of sucrose treatment. Thus, exogenous sucrose may inhibit miR399 expression and induce the expression of miR399-target genes, *SmNINV3* and *SmNINV4*, in *S. miltiorrhiza*.

## 3. Discussion

To date, the genome sequence [44,55] as well as a large number of transcriptomic sequences [41,56,57,58] are available for *S. miltiorrhiza*. These allow us to systematically analyze the alkaline/ neutral invertase gene family in *S. miltiorrhiza.* In this study, a total of nine *SmNINV* genes were identified and characterized. They were classified into two groups, including the α group and the β group. Subcellular localization prediction showed that α group SmNINVs, including SmNINV1–SmNINV4, contained a chloroplast transit peptide. No N-terminal presequence was found for β group SmNINVs, including SmNINV5–SmNINV9. The results are consistent with previous studies [13]. Different localizations exhibited by NINVs may grant these enzymes important physiological roles. The miR399-regulated SmNINVs, SmNINV3, and SmNINV4, are both chloroplast-localized. Since the chloroplast-localized AtNINV participates in controlling chloroplast-cytosolic carbon partitioning [13], miR399 may be also involved in the control of carbon balance between cytosol and chloroplasts through regulating SmNINV3 and SmNINV4. In addition, differences between α group SmNINVs and β group SmNINVs were found in motif distribution and exon-intron structure. It indicates that the members of α and β groups are probably evolved from different ancestors [5,13].

It has been shown that AT1G35580 and AT4G09510, two closely related *Arabidopsis* alkaline/ neutral invertase isoforms in β2 subgroup, play vital roles in primary root development [16,25,26]. The other β2 subgroup member, tea CsNINV14, was responsive to drought, NaCl and ABA treatments. Under drought condition, the expression of *CsNINV14* was up-regulated in tea leaves and down-regulated in roots [23]. In this study, we found that *SmNINV8*, which showed close phylogenetic relationships with *AT1G35580*, *AT4G09510* and *CsNINV14*, exhibited high expression in roots, particularly in root xylem, and responded to drought and MeJA treatments. It indicates the significance of *SmNINV8* in plant defense and root development. *AT3G05820*, a member of the α1 subgroup, was mainly expressed in reproductive tissues [29]. Loss-of-function of *AT3G05820* resulted in severely reduced shoot growth and delayed flowering [29]. *SmNINV1*, a *SmNINV* closely related to *AT3G05820*, was mainly expressed in flowers as well. It indicates that *SmNINV1* may be also involved in flowering and shoot development. Additionally, we found that stress-responsive *SmNINVs* mainly belonged to β group in the NJ phylogenetic tree. In order to make clear whether the α group NINVs participate in plant stress management, we searched stress-responsive NINVs in other plants. In *Populus*, two of the α group NINVs, *PtrNINV3* and *PtrNINV4*, were upregulated in leaves and roots under salt stress but downregulated in leaves under cold stress and pathogenic bacteria infection [59]. The results indicated that the α group NINVs can be stress-responsive as well. Since most genes are involved in stress responses in a tissue- and time-specific manner, the α group *SmNINVs*, including *SmNINV1*-*SmNINV4*, may participate in stress management at specific tissues and treatments. Further analysis through genetic transformation will help to verify the function of *SmNINVs* in *S. miltiorrhiza*.

It has been shown that miR399 is involved in plant responses to the starvation of nitrogen (N) and other nutrients, such as Fe, K, Na, and Ca, and plays a key role in the signaling network of Pi starvation responses in *Arabidopsis* through cleaving *PHO2* transcripts, which encode a ubiquitin-conjugating E2 enzyme [33,34,35,36,37,60,61]. Pi starvation induced the expression of miR399 in *Arabidopsis* shoots and roots. Concomitantly, its target, *PHO2*, was down-regulated [33,34,35,36,60]. In transgenic *Arabidopsis* overexpressing miR399 and in *pho2* T-DNA knockout plants, the expression of *PHO2* transcripts was suppressed in accordance with the accumulation of excessive Pi in shoots [33,60]. In this study, we found that Smi-miR399 targets *SmPHO2* for cleavage. It suggests that the miR399-*PHO2* module is conserved in *S. miltiorrhiza*.

**Figure 12:**
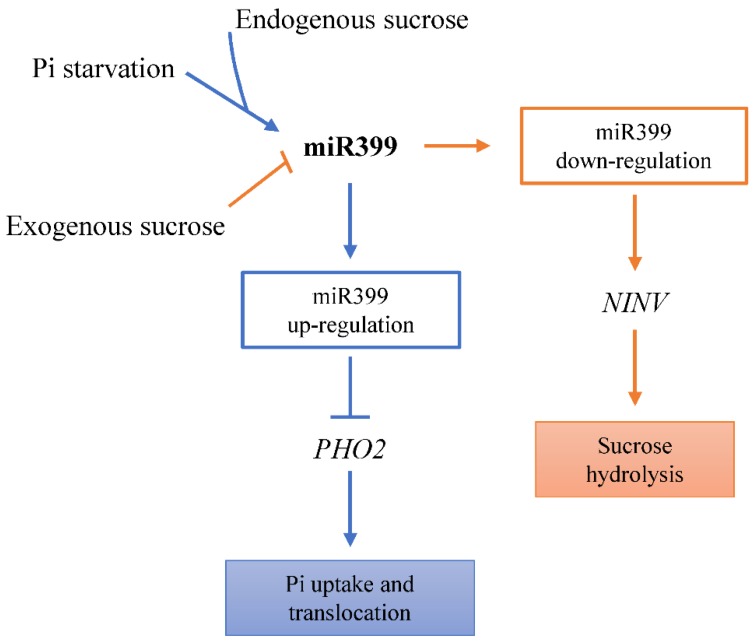
Integration of the miR399-*PHO2* pathway and the miR399-*NINV* pathway in plants. In the miR399-*PHO2* pathway (blue), Pi starvation and endogenous sucrose induce the expression of miR399, which further improves Pi uptake and translocation through down-regulation of *PHO2*. In the miR399-*NINV* pathway (brown), exogenous sucrose inhibits the expression of miR399, resulting in up-regulation of NINVs and sucrose hydrolysis. MiR399 acts as an integrator of the two pathways.

In addition, the expression level of miR399 can also be affected by sucrose. Previous studies showed that higher endogenous sucrose accumulation was accompanied by higher miR399 expression, whereas exogenous sucrose supplementation inhibited miR399 accumulation under phosphate starvation conditions [53]. In this study, we found the existence of the miR399-*NINV* module in various plant species (Table 2). We experimentally proved that both miR399 and *NINVs* were sucrose-responsive. Exogenous sucrose inhibited miR399 expression and induced the expression of miR399-target genes, *SmNINV3* and *SmNINV4*, in *S. miltiorrhiza*. It suggests that miR399, *SmNINV3* and *SmNINV4* may be crucial for sucrose metabolism in *S. miltiorrhiza*. Given that miR399 was also up-regulated by Pi starvation and promoted by photosynthetic carbon assimilation during the onset of Pi starvation [62], we proposed the existence of a cross-talk among phosphate homeostasis, sucrose signaling and miR399, in which miR399 acted as an integrator of phosphate homeostasis and sucrose signaling. The integration can be accomplished through the cleavage of *PHO2* and *NINV* transcripts (Figure 12). Our results are benefical to establish a novel link between miR399 and *NINVs* in plants.

## 4. Conclusions

*NINVs* are important to carbohydrate metabolism and plant development. In this study, we identified and characterized nine *SmNINV* genes from a well-known TCM plant, *Salvia miltiorrhiza*. Phylogenetic analysis showed that SmNINV proteins could be classified into two groups, including the α group and the β group. Four SmNINVs, including SmNINV1–SmNINV4, belong to the α group, whereas the other five SmNINVs, including SmNINV5–SmNINV9, are included in the β group. SmNINVs in different groups showed differences in motif distribution, exon-intron structure and subcellular localization and could have different ancestors. The α group SmNINVs contain a chloroplast transit peptide, whereas no N-terminal signal peptide was found for β group SmNINVs. In addition, *SmNINVs* were differentially expressed in organs and different root tissues and were responsive to drought, salicylic acid, yeast extract, and methyl jasmonate treatments. Two α group *SmNINVs*, including *SmNINV3* and *SmNINV4*, were posttranscriptionally regulated by the miR399 family, which has five members in *S. miltiorrhiza*. Analysis of miR399-directed cleavage of *NINV* transcripts in other plant species, including *A. thaliana*, *P. trichocarpa*, *M. esculenta,* and *S. lycopersicum*, showed conservation of the miR399-*NINV* module in plants. Exogenous sucrose may inhibit miR399 expression and induce *SmNINV3* and *SmNINV4* expression in *S. miltiorrhiza*. In addition, the Pi starvation-responsive miR399-*PHO2* module found in other plant species also exists in *S. miltiorrhiza*. It suggests that miR399 may act as a significant integrator in phosphate homeostasis and sucrose signaling through regulating the expression of *PHO2* and *NINVs* in plants.

## 5. Materials and Methods

### 5.1. Plant Materials and Growth Conditions

The roots, stems, leaves and flowers of *S. miltiorrhiza* (line 99-3) plants were collected from the experimental field at the Institute of Medicinal Plant Development and immediately stored in liquid nitrogen until use. Three independent biological replicates were carried out for each organ. For sucrose treatment, leaves were treated with 3% sucrose for 3 h, 12 h, and 24 h and collected as described previously [53]. The details are as follows. First, the plantlets were transferred to liquid Hoagland’s medium for 2 days at 25 °C under a photoperiod of 16 h light and 8 h dark to adapt to the environment. Then, they were transferred in the dark for 24 h to be starved of sucrose. At last, the seedlings were transferred to Hoagland’s medium containing either 3% sucrose (treated samples, Sucrose) or without sucrose (controls, CK) in the dark. Hoagland’s medium were renewed daily to ensure pH stability. Each treatment was repeated for three times. Controls and treated samples were collected at the same time (3 h, 12 h and 24 h), then immediately stored in liquid nitrogen until use. Three biological replicates were performed.

### 5.2. SmNINV Gene Identification

The *Arabidopsis* alkaline/neutral invertase protein sequences were downloaded from the TAIR database (https://www.arabidopsis.org/). To predict *SmNINV* genes, *Arabidopsis* alkaline/neutral invertase protein sequences were used as queries to search the assembly of *S. miltiorrhiza* (line 99–3) whole genome sequence [43] using the tBLASTn program with an expected value (e-value) cut off of 0.01. The gene models of the retrieved *SmNINV* candidates were examined by comparison with *NINV* genes identified in other plant species using the BLASTx algorithm (http://www.ncbi.nlm.nih.gov/BLAST) and manually corrected by alignment with *S. miltiorrhiza* transcriptome data (http://www.ncbi.nlm.nih.gov/sra). The intron/exon structures of *SmNINVs* were analyzed on the Gene Structure Display Server (GSDS 2.0, http://gsds.cbi.pku.edu.cn/index.php). Amino acid number, molecular weight (MW) and theoretical isoelectric point (p*I*) were analyzed on the EXPASY server (http://www.expasy.org/tools/protparam.html). Conserved domains in *SmNINV* proteins were searched against the Pfam protein family database (http://pfam.xfam.org/). Conserved motifs were predicted using the MEME suite (http://meme.sdsc.edu/meme/meme.html). Subcellular localization of SmNINVs and AtNINVs was predicted on the TargetP1.1 server (http://www.cbs.dtu.dk/services/TargetP/).

### 5.3. Sequence Alignment and Phylogenetic Analysis

All of the amino acids of SmNINVs were analyzed using DNAman 6.0 software (Lynnon Biosoft, Quebec City, QC, Canada). The substrate-binding residues and catalytic residues were depicted based on the results from CaNINVs [44]. The neighbor-joining (NJ) tree was constructed using MEGA version 7.0 [45]. Bootstrap tests were performed using 1000 replicates to assess the confidence of the phylogenetic relationships.

### 5.4. RNA Extraction and qRT-PCR Analysis

Total RNA and miRNA were extracted from organ samples using the EASYspin Plus Plant RNA Kit (Aidlab, Beijing, China) and the EASYspin Plant microRNA kit (Aidlab, China), respectively. RNA quantity was evaluated using NanoDrop 2000C Spectrophotometer (Thermo Scientific, Waltham, MA, USA). RNA integrity was evaluated using a 1.2% agarose gel. Reverse transcription of total RNA was performed using the PrimeScript™ RT reagent kit with gDNA Eraser (Perfect Real Time) (TaKaRa, Dalian, China). Gene specific primers were designed on NCBI (https://www.ncbi.nlm.nih.gov/tools/primer-blast/) (Table S6). *SmUBQ10* was used as a reference gene. Reverse transcription of miRNA was performed using Mir-X miRNA First-strand synthesis Kit (Takara, Dalian, China). The primers were listed in Table S6. The qRT-PCR analysis was conducted in triplicate using TB Green™ Premix Ex Taq™ II (Tli RNaseH Plus) (TaKaRa, Dalian, China) on a CFX96^TM^ real-time PCR detection system (Bio-Rad, Hercules, CA, USA). Relative gene expression was analyzed using the 2^−ΔΔCt^ method [63]. One-way ANOVA was performed using IBM SPSS 20 software to detect differential transcript abundance among organs and treatments. 

### 5.5. RNA-seq Data and Bioinformatic Analysis

Transcriptome sequencing datasets from roots, stems, leaves and flowers of *S. miltiorrhiza* were downloaded from the SRA database under the accession numbers SRP051564 and SRP028388. RNA-seq reads from root periderm, root phloem and root xylem of *S. miltiorrhiza* were downloaded from the SRA database under the accession number SRR1640458. RNA-seq reads from *S. miltiorrhiza* treated with drought stress were downloaded from the SRA database under the accession numbers SRR6813611 and SRR6813609. RNA-seq reads from salicylic acid (SA)-treated leaf callus cell culture of *S. miltiorrhiza* were downloaded from the SRA database under the accession number SRX1423774. RNA-seq data from *S. miltiorrhiza* hairy roots treated with yeast extract (YE) and methyl jasmonate (MeJA) were downloaded from the SRA database under the accession number SRP111399. Differential gene expression of *SmNINVs* in various organs, root tissues and under treatments was analyzed using SOAP 2.0 as described previously [64,65]. *SmNINV* gene expression level was estimated by reads per kilobase per million mapped reads (RPKM). Heatmaps were created using the software package on the BMKCloud online server (http://www.biocloud.net/).

### 5.6. Identification of S. miltiorrhiza miRNAs Potentially Targeting SmNINVs 

*S. miltiorrhiza* miRNAs potentially targeting *SmNINVs* were predicted using psRNATarget (http://plantgrn.noble.org/psRNATarget/) with the maximum expectations of 3.0. All of the candidate miRNAs potentially targeting *SmNINVs* were mapped to the assembly of *S. miltiorrhiza* (line 99–3) whole genome sequence [43] using the psRobot software package with the default parameters [48]. The resulting hairpin structures were checked manually according to the criteria described previously [49]. Computational validation of miRNA-directed cleavage was carried out through the analysis of degradome data.

### 5.7. 5′ RLM-RACE Validation of miR399-Directed Cleavage

Experimental validation of miR399-directed cleavage was carried out on mRNA isolated from roots, stems, leaves and flowers of *S. miltiorrhiza* using the modified 5′ RNA ligase-mediated (RLM)-RACE method as described previously [52]. The nesting and nested primers used for PCR were listed in Table S7.

## Figures and Tables

**Table 1:** Sequence features of *alkaline*/*neutral invertase* (*NINV*) genes in *S. miltiorrhiza*.

Gene Name	Gene Length (bp)	ORF Length (bp)	Protein Length (aa)	MW (kDa)	No. of Intron	*p*I	Loc ^a^	TPlen ^b^
*SmNINV1*	3088	1962	653	74.09	4	6.55	C	8
*SmNINV2*	2535	2028	675	76.15	4	5.99	C	34
*SmNINV3*	2954	1923	640	72.17	5	6.51	C	47
*SmNINV4*	2843	1851	616	69.86	5	8.67	C	44
*SmNINV5*	2796	1818	605	67.6	3	5.53	-	-
*SmNINV6*	2648	1731	576	65.68	3	6.09	-	-
*SmNINV7*	3112	1704	567	64.72	3	6.2	-	-
*SmNINV8*	2701	1710	569	64.61	3	6.02	-	-
*SmNINV9*	2395	1713	570	64.73	3	6.41	-	-

^a^ Loc represents the protein localization predicted by TargetP1.1. ‘C’, which stands for chloroplast, suggests that the sequence contains a chloroplast transit peptide. ‘-’ indicates any locations other than the chloroplasts, the mitochondria and the secretory pathways. ^b^ TPlen represents the length of predicted signal peptide sequence.

**Table 2:** The miR399-*NINV* module in *S. miltiorrhiza*, *A. thaliana*, *P. trichocarpa*, *M. esculenta*, and *S. lycopersicum*.

Plant Species	miR399 Members Involved	miR399-Regulated *NINVs*	Methods Used for Analysis
*S. miltiorrhiza*	Smi-miR399a–Smi-miR399e	*SmNINV3* and *SmNINV4*	Computational prediction, degradome analysis, and 5’ RLM-RACE validation
*A. thaliana*	Ath-miR399b, Ath-miR399c	*AT1G56560* and *AT3G06500*	Computational prediction and degradome analysis
*P. trichocarpa*	Ptc-miR399i	*PtNINV3*, *PtNINV4*, and *PtNINV6*	Computational prediction
*M. esculenta*	Mes-miR399f	*MeNINV6*, *MeNINV7*, and *MeNINV10*	Computational prediction
*S. lycopersicum*	Sly-miR399	*SlNINV1*	Computational prediction

## Supplementary Materials

The following are available online at https://www.mdpi.com/2223-7747/8/11/490/s1. Table S1: Expression levels of *SmNINV* genes in different organs (RPKM); Table S2: Expression levels of *SmNINV* genes in different root tissues (RPKM); Table S3: Expression of *SmNINVs* in response to drought (RPKM); Table S4: Expression of *SmNINVs* in response to salicylic acid (SA) treatment (RPKM); Table S5: Expression of *SmNINVs* in response to yeast extract (YE) and methyl jasmonate (MeJA) treatments (RPKM); Table S6: Primers used for qRT-PCR; Table S7: Primers used for 5’ RLM-RACE.
